# Supplementary material for: Remote Eradication of Bacteria on Orthopedic Implants via Delayed Delivery of Polycaprolactone Stabilized Polyvinylpyrrolidone Iodine
Source: J Funct Biomater. 2022 Oct 19;13(4):195. doi: 10.3390/jfb13040195 (PMC9589933; doi:10.3390/jfb13040195)
Supplement: Supplementary file 1 [file jfb-13-00195-s001.zip › jfb-1956474-supplementary.pdf]

Supplementary Materials

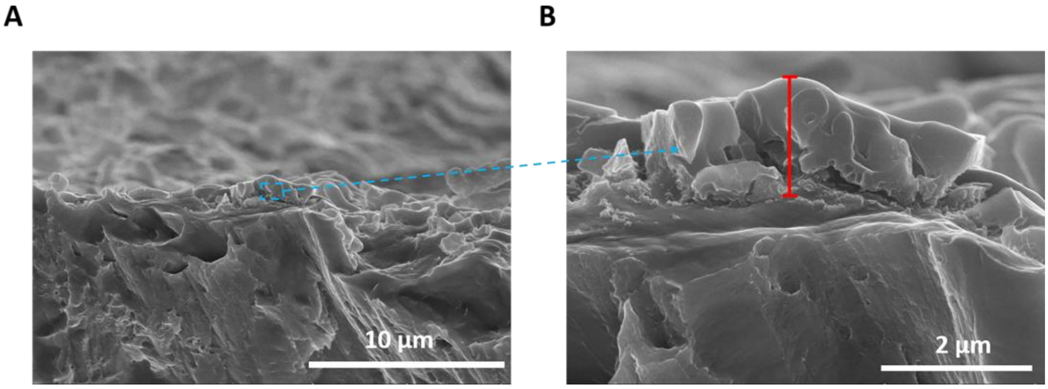

Figure S1. Cross-sectional SEM morphology of the MAO coating. (A) 5000× magnification of the MAO cross-section SEM image ;(B) 20,000× magnification of the MAO cross-section SEM image, the thickness of the MAO coating is about 2 µm.

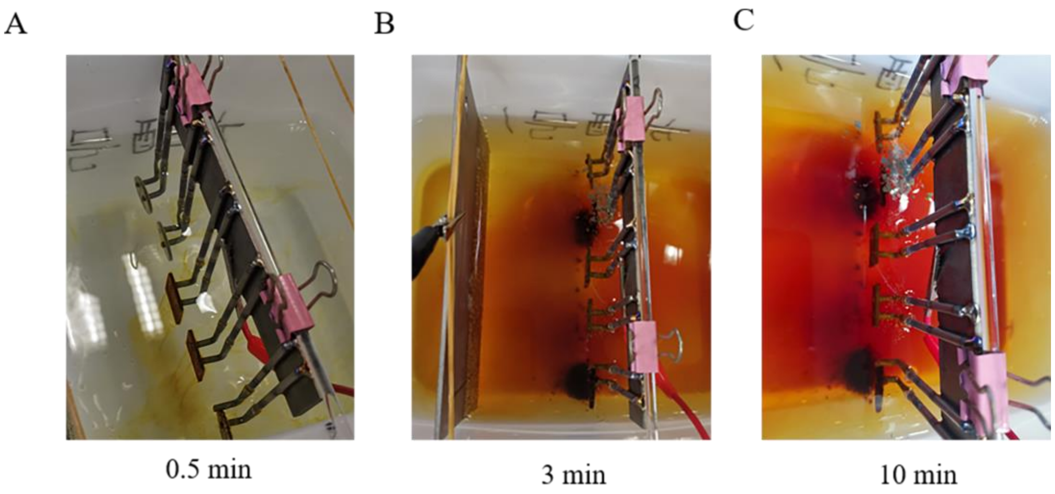

Figure S2. Iodine-loaded electrophoresis process. (A–C) Scenarios of electrophoretic loading of iodine with time.

Table S1. Composition of anodic oxidation electrolytic solution.

**Anodic oxidation electrolytic solution**		
Substance	Concentration	Purity
H_2SO_4	35g/L	Analytic reagent
H_3PO_4	25g/L	Analytic reagent
H_2O_2	10g/L	Analytic reagent
ddH_2O	The capacity to 1L	Magnetic stirring

Table S2. Composition of electrophoretic solution.

**Electrophoretic solution**		
Substance	Concentration	Purity
KI	1 wt%	Analytic reagent

PVPI	1 wt%	Analytic reagent
ddH_2O	The capacity to 1L	Magnetic stirring
